# Exploring the pore charge dependence of K^+^ and Cl^−^ permeation across a graphene monolayer: a molecular dynamics study†

**DOI:** 10.1039/c9ra03025e

**Published:** 2019-07-01

**Authors:** Carlo Guardiani, William A. T. Gibby, Miraslau L. Barabash, Dmitry G. Luchinsky, Peter V. E. McClintock

**Affiliations:** Department of Physics, Lancaster University Lancaster LA1 4YB UK c.guardiani@lancaster.ac.uk +44 (0)1524 844037 +44 (0)1524 593073; SGT Inc. Greenbelt MD 20770 USA

## Abstract

Selective permeation through graphene nanopores is attracting increasing interest as an efficient and cost-effective technique for water desalination and purification. In this work, using umbrella sampling and molecular dynamics simulations with constant electric field, we analyze the influence of pore charge on potassium and chloride ion permeation. As pore charge is increased, the barrier of the potential of mean force (PMF) gradually decreases until it turns into a well split in two subminima. While in the case of K^+^ this pattern can be explained as an increasing electrostatic compensation of the desolvation cost, in the case of Cl^−^ the pattern can be attributed to the accumulation of a concentration polarization layer of potassium ions screening pore charge. The analysis of potassium PMFs in terms of forces revealed a conflicting influence on permeation of van der Waals and electrostatic forces that both undergo an inversion of their direction as pore charge is increased. Even if the most important transition involves the interplay between the electrostatic forces exerted by graphene and water, the simulations also revealed an important role of the changing distribution of potassium and chloride ions. The influence of pore charge on the orientation of water molecules was also found to affect the van der Waals forces they exert on potassium.

## Abstract 2

Increase of graphene pore charge determines decrease of PMF barrier that turns into well: current increases, reaches plateau and declines.

## Graphical abstract

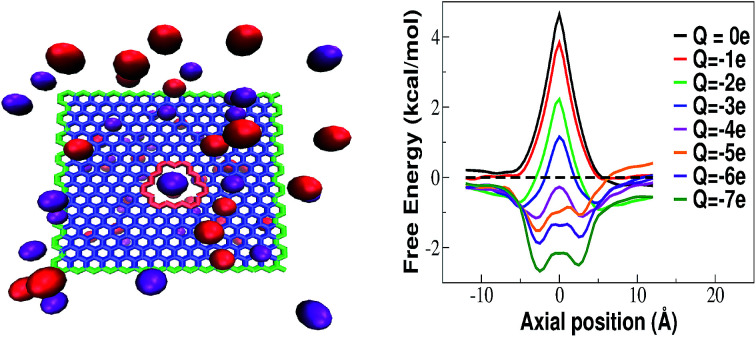

## Introduction

Graphene is a thin membrane consisting of sp^2^-bonded carbon atoms arranged in a honeycomb lattice.^1^ Due to its peculiar structure, graphene is endowed with excellent thermal^2^ and electric conduction properties^3^ which make it widely used in energy storage devices like supercapacitors^4^ and Li-ion batteries.^5^ Moreover, using electron beam irradiation^6^ or block copolymer lithography^7^ it is now possible to drill nanoscale pores in a single graphene layer. This technology paves the way to a wide range of potential applications in the fields of desalination of seawater,^8^ wastewater purification^9^ and DNA sequencing.^10^ These applications take advantage of the ultra-thinness of graphene which allows fast water transport while excluding ions or selecting specific ion types. The performance of these technologies critically relies on precise control of pore size. In fact, atomically precise pore sizes would allow the development of molecular sieves capable of separating compounds with atomic scale differences in size and shape. Significant advances in this field have been achieved by Gilbert *et al.*^11^ who demonstrated the fabrication of individual nanopores in hexagonal boron nitride with atomically precise control of pore shape and size, and by Thirumaran *et al.*^12^ who, using controlled Ga^+^ ion irradiation, introduced a population of sub-nanometer pores in a MoS_2_ membrane, realizing atomic transport measurements. Despite these recent achievements, experiments on transport across nanopores in 2D materials such as graphene, boron nitride, molybdenum disulfide and tungsten disulfide, are still lacking and most studies infer the conductance and sub-nanometer pore diameters indirectly from computational modelling.

In a pioneering Molecular Dynamics (MD) study Cohen-Tanugi *et al.*^13^ showed that for small nanopores the sieving effect can be ascribed to steric hindrance of the pore edge. More specifically, it was shown that in order to effectively exclude salt ions, the diameter of a graphene nanopore cannot exceed 5.5 Å. Since the creation of small nanopores is technologically more challenging than drilling larger ones,^14,15^ alternative selectivity strategies suitable for larger pores have been actively sought. In particular, it was shown that placement of charges on the pore edge by chemical functionalization makes the pore selective to counter-ions. Using this strategy Sint *et al.*^8^ showed that replacement of carbon atoms of the graphene pore with nitrogen and fluorine makes the pore cation selective while addition of hydrogen make it anion selective. Moreover, Zhao *et al.*^16^ showed computationally that even when the pore radius is much larger than the hydrated radius of the ion, negatively charged nanopores still exhibit remarkable selectivity. This finding was confirmed by Konatham *et al.*^17^ that however observed that the selectivity induced by charged groups becomes less effective as the pore diameter is increased.

The main research question addressed by the many research groups working on graphene nanopores is the following: what is the ideal functionalization providing high water flows while discriminating against specific solutes? Biological ion channels, shaped through millions of years of evolution, are characterized by flow rates comparable to the free diffusion limit while retaining high selectivity.^18^ For instance, K^+^ channels like KcsA pass potassium and sodium with a ratio 1000 : 1 (ref. 19) while Na^+^ channels select sodium over potassium with an efficiency up to 100 : 1.^20^ Biological ion channels, cannot be used directly in technological applications due to their very poor mechanical properties and a tendency to lose their function after leaving the biological environment. However, they represent a great source of inspiration to design artificial nanopores. Kang *et al.*^21^ for instance, designed oxygen doped graphene nanopores showing that selectivity to K^+^ over Na^+^ can be achieved if the distance between oxygens replicates that observed in the Selectivity Filter (SF) of KcsA. A similar approach was employed by He *et al.*^22^ who designed bio-inspired graphene nanopores containing four carbonyl groups (4CO) mimicking KcsA SF, or four carboxylate groups (4COO) arranged as in the SF of the NavAb Na^+^ channel.

The design principles of biological ion channels, however, are far from being completely understood. For instance, MD simulations showed^22^ that the 4CO construct by He *et al.*, as expected, was potassium selective. Surprisingly however, the 4COO construct that was expected to mimick the properties of NavAb, was not sodium but potassium selective and the selectivity of another construct with three carboxylate groups turned out to be voltage-tunable. Moreover, when the pore diameter is less than 5 nm, the confined liquid structure in the nanopore affects various transport properties of the ions.^23^ Since such properties may deviate from the bulk medium behaviour, ion dynamics in a nanopore confined region or an atomically thin membrane is still not well understood. Another open problem is related to the fact that functionalization can lead to a high density of charge along the pore rim. This in turn, leads to the accumulation of a concentration polarization layer in the neighborhood of the graphene membrane.^24^ The influence of this Debye layer on ion current is still not clear.

In this paper we study the dependence on pore charge of K^+^ and Cl^−^ ion permeation through a pore drilled in a graphene monolayer. This enabled us to identify general trends useful for the design of functionalized nanopores. The current–voltage curves derived from MD simulations in the presence of a constant electric field were analyzed in terms of the potential of mean force (PMF) computed through umbrella sampling (US) simulations.^25^ We discovered that, as pore charge is increased, the barrier of K^+^ PMF gradually decreases until it turns into a well split in two sub-minima. An analysis of electrostatic energy and desolvation of the ion biased in US simulations, showed that the approximately constant desolvation cost is better and better compensated by the energy of electrostatic interaction with the charged pore. Thus this scenario, appears to be consistent with theoretical models by Eisenman^26,27^ and Zwolak.^28,29^ However, the analysis of the forces acting on the biased ion revealed a richer picture where a relevant role is also played by the potassium and chloride ions whose tendency to accumulate in concentration polarization layers significantly affects the electrostatic forces. Also, we observed that pore charge influences the orientation of water molecules affecting the van der Waals force they exert on the solvated K^+^ ion. Finally, the study of Cl^−^ permeation revealed that, even if the PMF profiles are extremely powerful tools for the analysis of ion permeation, they must be used with extreme care. In fact, asymmetric ion distributions arising from the application of high voltages, cannot be accounted for by a PMF computed in the *V* = 0 regime, but they have a profound influence on ionic currents.

The paper is organized as follows. After the description of our computational methodology, we study potassium permeation devoting particular attention to the analysis of the forces and orientational effects that are discussed in specific subsections. The Results section concludes with the analysis of chloride permeation where a seeming discrepancy between *I*–*V* curves and PMF profiles is reconciled in terms of the effect of voltage-induced asymmetric ion distributions. Finally, we draw conclusions.

## Methods

### System set-up

**Fig. 1:**
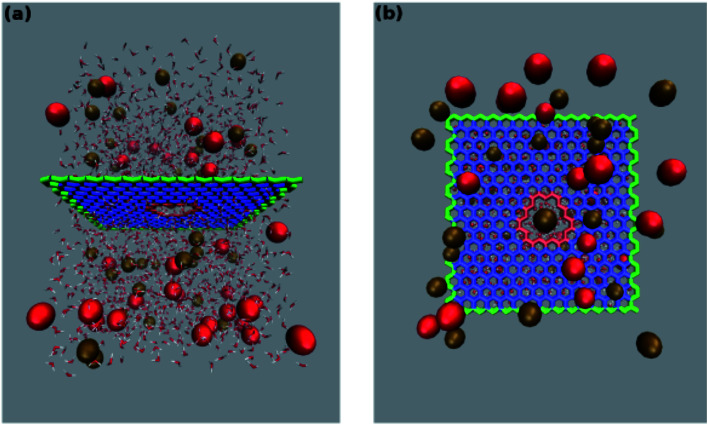
Configuration of the simulation system. Side (a) and top view (b) of the pore in a graphene monolayer bathed in a KCl solution. A charge ranging from −1*e* to −7*e* was uniformly spread over the rim atoms (highlighted in red) while a neutralizing positive charge was spread over all the other atoms of the graphene layer. The outermost graphene atoms (highlighted in green) were held fixed while all the other atoms were allowed to move freely. Potassium ions are portrayed as brown beads while red beads represent chloride ions. For the sake of clarity in (b) only the water molecules beyond the graphene layer are shown.

The system studied comprises a single graphene layer with a nanopore of radius *r*_p_ 4.5 Å. The pore is thus larger than those of typical biological ion channels. However, the radius is below the 5.5 Å cutoff for unselective ion transport^16^ so that permeation and selectivity are mainly determined by the rim charge. The pore was generated choosing a reference carbon atom with coordinates (*x*_r_, *y*_r_, *z*_r_) and removing all the graphene atoms whose distance from the reference atom 
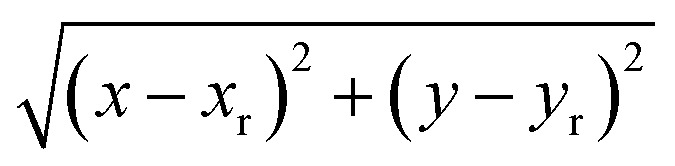
 was below the chosen pore radius. The pore was either left uncharged or assigned an overall charge −1*e*, −2*e*, −3*e*, −4*e*, −5*e*, −6*e* or −7*e*. The pore charge was evenly spread over all the atoms lining the pore rim (green atoms in Fig. 1). The neutrality of the system was enforced by spreading a counter-balancing positive charge over all the other atoms of the graphene sheet (red and blue atoms in Fig. 1). The graphene layer was bathed on both sides by a 1 M solution of KCl and the size of the simulation box was 35 × 35 × 50 Å^3^. The outermost atoms of the graphene sheet (green atoms in Fig. 1) were held fixed while all the other atoms were completely unconstrained. All simulations were performed with the NAMD 2.12-mp software^30^ using the CHARMM27 force field^31^ and the TIP3P water model.^32^ The equilibration, in the NPT ensemble at 300 K and 1 atm, was organized in three stages with increasing time-steps (0.5, 1.0 and 2.0 fs) run for 0.5, 1.0 and 2.0 ns respectively. Each equilibration stage was preceded by 1000 steps of conjugate gradient minimization. The short-range van der Waals and electrostatic interactions were cut off at 12.0 Å with a switching distance of 10.0 Å. The long-range electrostatic interactions were computed with the particle mesh Ewald method. Periodic boundary conditions were imposed in all directions and coordinates were stored every 2 ps.

### Simulations under external voltage

After equilibration the system was simulated in the presence of a uniform electric field^33^ corresponding to potential differences ranging from −0.5 to −4 volts at 0.5 V intervals. The constant field was computed as *E*_*z*_ = *V*/*L*_*z*_ where *V* is the desired potential difference, while *L*_*z*_ is the length of the simulation box along the *z*-axis. Following ref. 33 and 34 the current was computed as: 
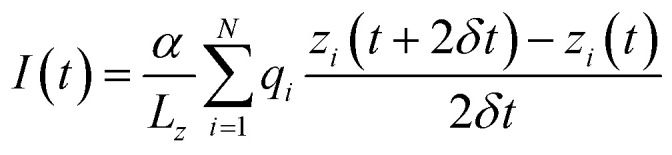
where the sum runs over all the ions of a given type, *L*_*z*_ is the length of the simulation box in the direction of the channel axis, *α* is a conversion factor to express the current in amperes, *q*_*i*_ and *z*_*i*_ are the charge and position of ion *i* and the displacement is computed over two sampling intervals *δt*. In order to perform a statistical analysis through block-averages, the 40 ns long trajectories, run in the NVT ensemble at the temperature of 300 K, were split in 4 10 ns-blocks. Currents are computed as averages, and errors as standard deviations over the four blocks.

### Potential of mean force calculation

In order to obtain the potential of mean force (PMF) of the permeating potassium ion the umbrella sampling method^25^ was employed. In these simulations the axial coordinate *z* of a single K^+^ was harmonically restrained (with force constant 1.0 kcal mol^−1^ Å^−2^) while the radial position was subject to a semi-harmonic wall at 4.5 Å from the pore axis. We considered 81 axial windows from *z* = −20.0 Å to 20.0 Å of width 0.5 Å. In the calculation of the K^+^ PMF each window was run for 6.0 ns with the first 1.0 ns considered as equilibration and excluded from the analysis. In the calculation of the Cl^−^ PMF, due to slower convergence, each window was run for 16 ns with the first nanosecond discarded as equilibration. Using the implementation by Grossfield^35^ the Weighted Histogram Analysis Method (WHAM)^36^ was used to calculate the PMF with a tolerance of 10^−7^.

## Results

### Analysis of potassium permeation

**Fig. 2:**
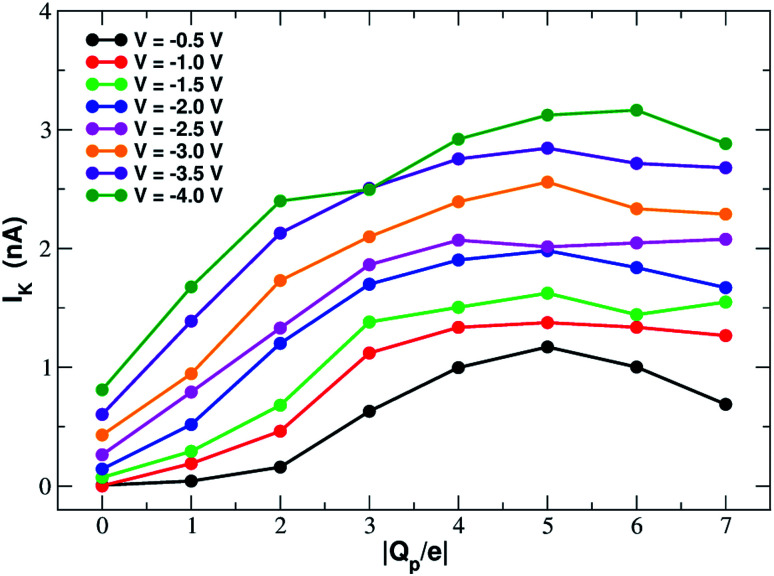
Potassium current as a function of the charge of the graphene pore.

**Fig. 3:**
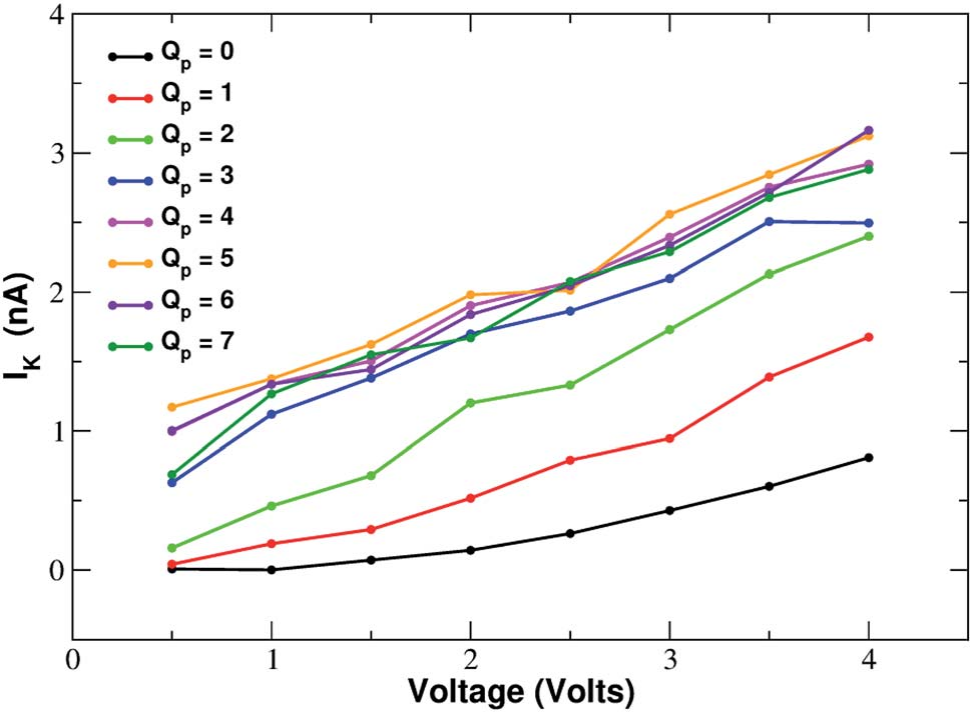
Current–voltage relationship of potassium in charged graphene monolayers with a pore charge ranging from *Q*_p_ = 0 to *Q*_p_ = −7. For graphical clarity the *x*-axis shows the absolute value of the negative voltages used in the simulations. Charges are expressed in elementary charge units.

In order to assess how pore charge affects K^+^ permeation, we ran MD simulations with an external applied potential in the range from −0.5 V to −4.0 V at 0.5 V intervals (Fig. 2 and 3). The profile of potassium current as a function of the pore charge is illustrated in Fig. 2. It can be noted that, as the pore charge is increased, the potassium current *I*_K_ increases at all voltages in a very steep way up to *Q*_p_ = −3*e* or *Q*_p_ = −4*e*. *I*_K_ then remains almost constant or slowly increases up to *Q*_p_ = −5*e* and then it drops for higher pore charges.

As will become more clear from the PMF calculations detailed later on, the behaviour of *I*_K_ as a function of *Q*_p_ can be explained as follows. When the pore is neutral the potassium ion is faced with a high desolvation barrier and only small currents can be recorded. As the pore charge is increased, the desolvation cost is partly balanced by the energy of electrostatic interaction with the charged pore. As a result, the barrier is decreased and the current increases. When the pore charge becomes very high, however, the barrier turns into a well where potassium ions are trapped for some time before being able to cross the pore, so that the current decreases.

**Fig. 4:**
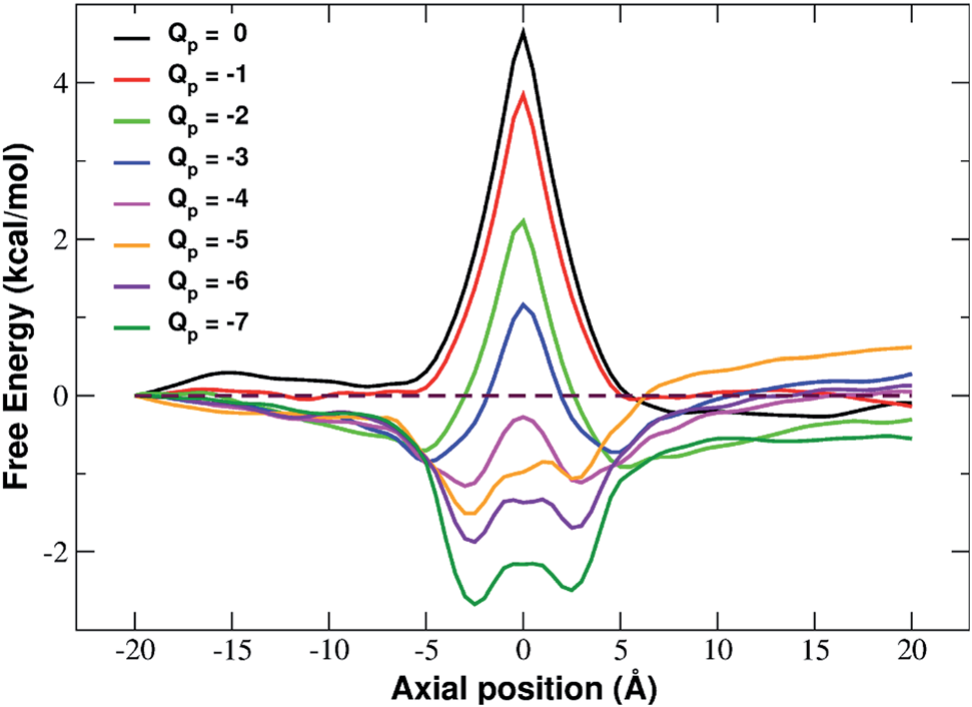
Potential of mean force of potassium as a function of the axial position. The calculation has been repeated for differently charged systems with pore charge ranging from *Q*_p_ = 0 to *Q*_p_ = −7. Charges are expressed in elementary charge units.

In fact, the PMF profiles reported in Fig. 4 show a high free energy barrier in the middle of the pore whose height decreases from 4.7 kcal mol^−1^ at *Q*_p_ = 0 to 1.05 kcal mol^−1^ at *Q*_p_ = −3*e*. For larger pore charges the barrier turns into a well of increasing depth split in two sub-minima centered at *z* = −2.5 Å and *z* = 2.5 Å.

**Fig. 5:**
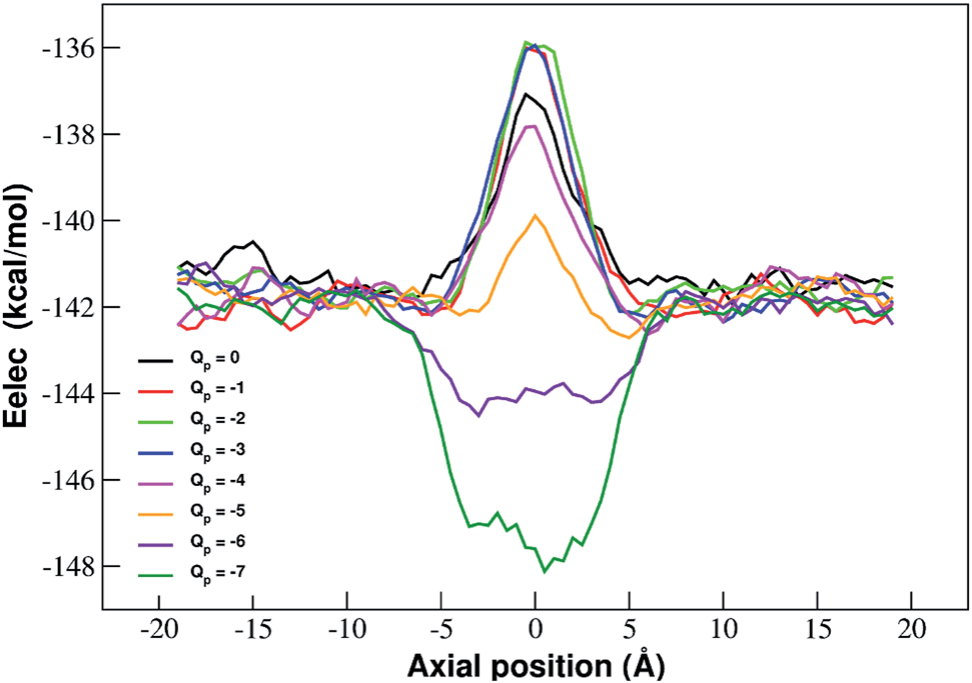
Profiles of average electrostatic energy experienced by the biased ion in the umbrella sampling windows. The curves are smoothed by means of running averages with windows of 5 data points (2.5 Å). Charges are expressed in elementary charge units.

The interpretation of PMF profiles is aided by the analysis of the profiles of the average electrostatic energy perceived by the biased ion in each umbrella sampling window. The contribution of van der Waals energy can be neglected since it only corresponds to 5% of the coulombic term. The electrostatic energy thus well approximates the total potential energy of the ion. The profile of average electrostatic energy (Fig. 5) shows a peak in correspondence of the pore that turns into a well as the rim charge is increased in a way that approximately mirrors the behaviour of the PMF.

The presence of an electrostatic energy peak in the position of maximal attraction between the positively charged ion and the negatively charged rim can only be understood considering the massive desolvation that the ion undergoes when passing through the pore. The ion/water interactions are, in fact, basically interactions between a charged particle and the permanent dipoles of water molecules. Indeed the high barrier observed at rim charges in the range [0 : −5] shows that the desolvation penalty largely overwhelms the electrostatic attraction with the charged pore. Clearly, as the pore charge is increased, the ion/pore electrostatic interactions compensate larger and larger fractions of the desolvation cost, causing a decrease of the barrier. Finally, when the charge reaches values of −6 and −7 the ion/pore interaction becomes so strong that it becomes dominant, generating an energy minimum.

It is interesting to note that the electrostatic energy minimum at *Q*_p_ = −6 and *Q*_p_ = −7 appears to be split into two subminima. In fact, while *in vacuo* there would be a single minimum in the middle of the pore where the ion/rim electrostatic interactions are more intense, in solution the electrostatic energy is optimized close to but outside the pore where the ion/rim interaction is still strong and the hydration shells are sufficiently preserved to allow also high ion/water interactions. This leads to the appearance of two sub-minima on either sides of the pore. Finally, it can be noted that the correspondence between electrostatic profiles and PMF profiles is only partial. In fact, in the PMF profiles, the two minima on either side of the pore appear already at *Q*_p_ = −2 while in the electrostatic profile they only appear at *Q*_p_ = −5. This suggests that the PMF can capture energetic or entropic contributions not accounted for by simple electrostatics.

More details of the dehydration process can be obtained by computing the number of water molecules in the first two hydration shells surrounding a restrained K^+^ ion in the windows of the umbrella sampling simulations. The calculation has been performed by integrating the radial density functions of the distance between the constrained ion and the oxygen atoms of water molecules.

**Fig. 6:**
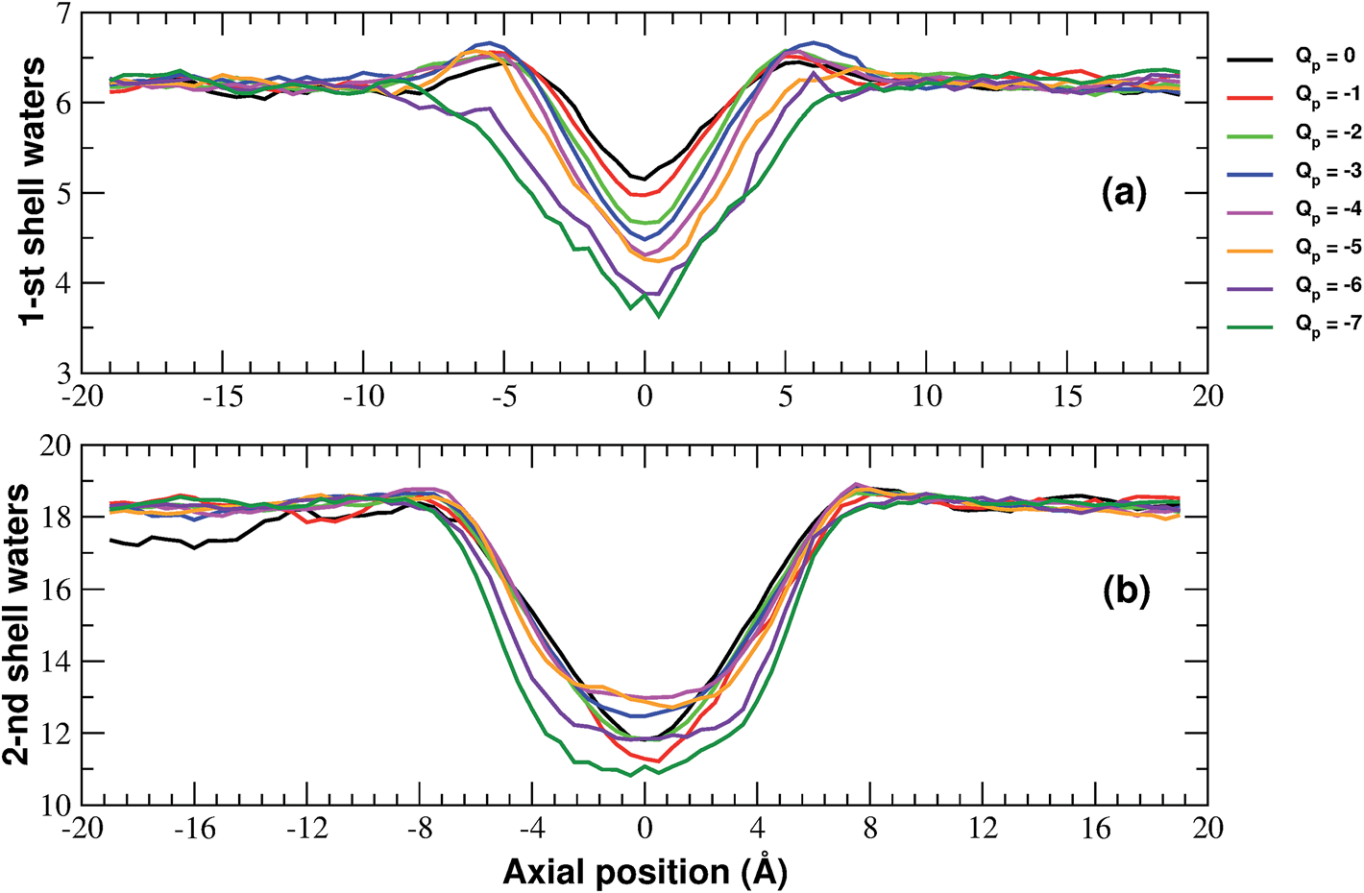
Numbers of water molecules in the first (a) and second (b) hydration shells of the potassium ion biased in the axial windows of the umbrella sampling simulation. The curves have been smoothed by means of running averages with windows of 5 data points (2.5 Å). Charges are expressed in elementary charge units.

As shown in Fig. 6, the second shell is massively desolvated resulting in a decrease in the number of water molecules from 18 to approximately 12. The first shell also experiences a loss of around 30% of its water even if in absolute terms it only loses 2 water molecules. Although the significant loss of water molecules occurring during the approach to the neutral pore highlights the importance of geometric factors, desolvation also appears to be affected by the pore charge.

In particular, Fig. 6(a) shows that the desolvation of the first shell is somewhat enhanced by the pore charge. This is possibly due to the fact that, with increasing rim charge, the ion in the pore becomes less and less mobile because the electrostatic attraction of the pore keeps it constrained in a region where there is only limited space for the water molecules of the hydration sphere.

The influence of pore charge on the desolvation of the second shell is more complex (Fig. 6(b)). The decrease of the water molecules tends to be attenuated as the pore charge is varied from −1 to −4 but is enhanced when the charge is further increased to *Q*_p_ = −6 or *Q*_p_ = −7. The scenario suggests that, while moderate values of the pore charge contribute to keep water molecules around the potassium ion, very high charge values cause a crowding of potassium ions around the pore, which limits the available space for the water molecules of the second shell.

### Analysis of forces

**Fig. 7:**
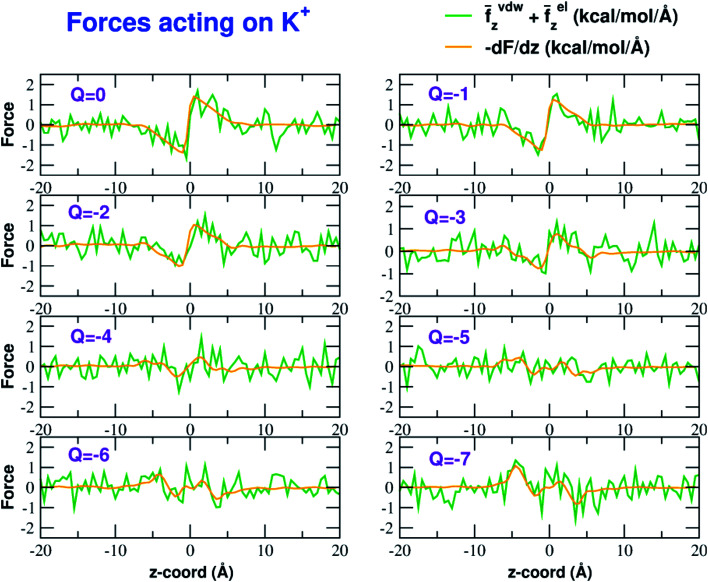
Forces acting on the K^+^ ion biased in the umbrella sampling simulations. The green line is the sum of the average *z*-components (orthogonal to the graphene layer) of the van der Waals and electrostatic forces exerted by all other atoms of the system. The orange line is the negative derivative of the axial potential of mean force *F*(*z*). Charges are expressed in elementary charge units.

**Fig. 8:**
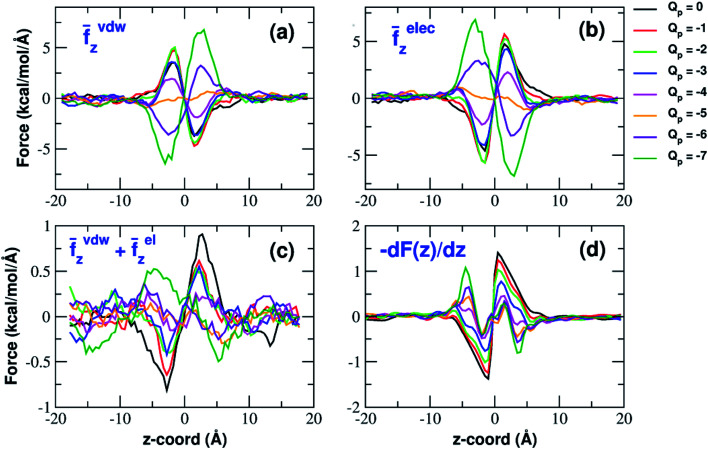
van der Waals and electrostatic forces acting on the K^+^ ion biased in umbrella sampling simulations. (a) Axial component of the average van der Waals force exerted by all other atoms of the system; (b) axial component of the electrostatic force exerted by the rest of the system; (c) sum of the *z*-components of van der Waals and electrostatic force. The curves have been smoothed by means of a running average with a 10-data-point moving window; (d) the negative derivative of the potential of mean force *F*(*z*). Charges are expressed in elementary charge units.

The influence of the pore charge on the PMF profile can be analyzed in terms of the average force acting on the potassium ion. Fig. 7 compares the sum of the average van der Waals and electrostatic forces acting on the K^+^ ion biased in umbrella sampling with the negative derivative of the PMF profile. The good agreement of the two curves suggests the average force to be an effective tool to get insights into the basis of the PMF shape. First of all, it can be noted that the van der Waals and electrostatic forces play opposing roles in influencing the motion of the ion towards the pore (Fig. 8). For instance, in the *z* < 0 region, the van der Waals force exhibits a positive peak whose height decreases and eventually turns into a deeper and deeper negative minimum as the pore charge is increased. In the *z* > 0 region the curve displays an anti-symmetric behaviour. This pattern shows that, for small values of the pore charge, the van der Waals force pushes the K^+^ ion towards the pore, whereas the ion is pulled towards the bulk for high values of *Q*_p_. By contrast, in the *z* < 0 region the electrostatic force profile forms a deep negative well that becomes shallower and shallower and finally turns into a positive peak of increasing height as the pore charge is increased. The anti-symmetric shape of the curve shows that the electrostatic force tends to pull the ion into the water phase but, as the pore charge is increased, the force is gradually reversed and pushes the ion towards the pore in the graphene layer.

**Fig. 9:**
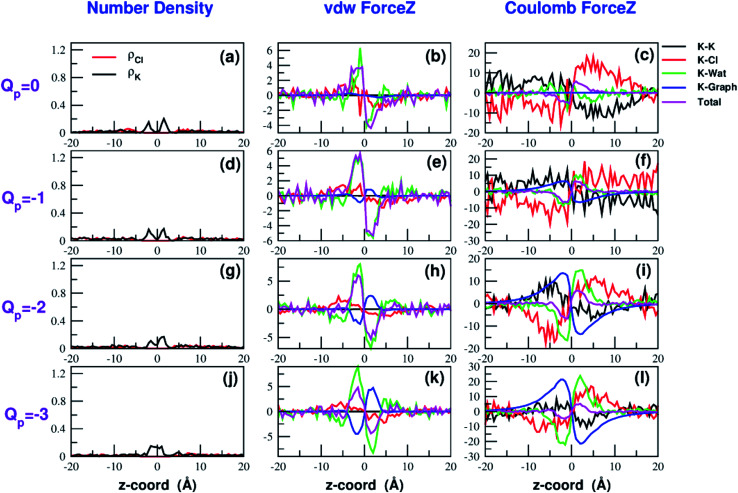
Breakdown of the van der Waals (b–k) and electrostatic forces (c–l) acting on the K^+^ ion biased in umbrella sampling simulations. The contribution of the other potassium ions is shown in black, that of chloride ions in red, that of water in green, that of graphene in blue and the total force is displayed in magenta. The first column (a–j) shows the number density profiles of potassium and chloride ions that affect the electrostatic forces. The four rows correspond to charges *Q*_p_ = 0 to *Q*_p_ = −3 of the graphene pore. Charges are expressed in elementary charge units.

**Fig. 10:**
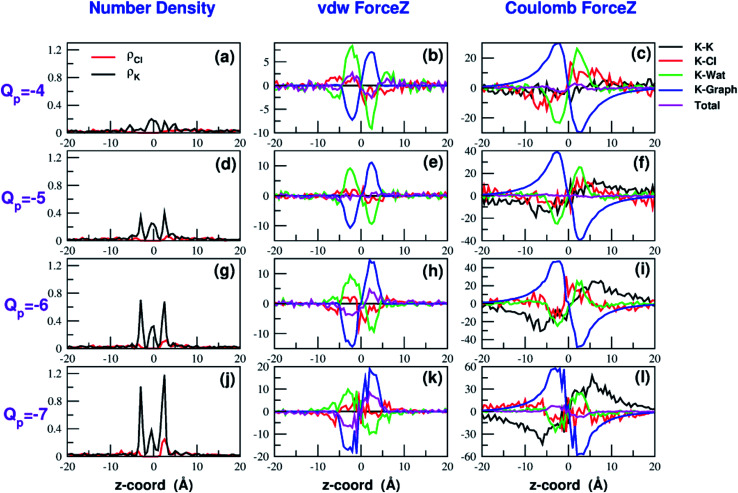
Continuation of Fig. 9. The four rows correspond to charges *Q*_p_ = −4 to *Q*_p_ = −7 of the graphene pore. Charges are expressed in elementary charge units.

Both the van der Waals and electrostatic forces acting on the K^+^ ion result from four contributions: (i) the force exerted by the other K^+^ ions; (ii) the force exerted by Cl^−^ ions; (iii) the force exerted by water molecules; (iv) the force exerted by graphene. Fig. 9 and 10 show the behaviour of the four force terms. Let us start from the electrostatic force that appears to be dominant compared to the van der Waals one. For small values of the pore charge most potassium and chloride ions are evenly dispersed in the bulk phase. As expected, the potassium ions repel the biased K^+^ pushing it away from the bulk and towards graphene. By contrast, the negatively charged chlorides attract the reference K^+^ ion pulling it into the bulk. Due to the 1 : 1 stoichiometry of KCl it is not surprizing that the forces exerted by potassium and chloride ions are almost symmetrical and tend to balance each other. However, at high values of the pore charge (*Q*_p_ values from −5 to −7) there is the appearance of a high local density of potassium ions on either side of the graphene layer. This local layer of potassium ions repels the reference K^+^ ion, pushing it away from graphene and reinforcing the effect of chloride ions. The force exerted by K^+^ and Cl^−^ ions is however, comparatively small with respect to that exerted by graphene and water. When the pore is neutral, graphene exerts a zero electrostatic force. However, as the pore charge is increased, graphene exerts a stronger and stronger attracting force on the reference K^+^ ion. Finally, the force exerted by water pulls the K^+^ ion strongly into the bulk. In summary, the reversal in the sign of the total electrostatic force occurs when the graphene force becomes dominant compared to the water force and the potassium force starts pushing the reference K^+^ ion into the bulk.

The van der Waals force also shows some interesting trends. First of all, note that graphene exerts a repulsive force, increasing with the pore charge. This is due to the fact that, as the pore charge is increased, the reference K^+^ ion is pulled closer and closer to the charged pore entering into the repulsive branch of the Lennard-Jones potential of the carbon atoms. Another notable feature of the van der Waals forces is that the force exerted on the reference K^+^ by the other potassium ions is always negligible. This is due to the fact that the repulsive K^+^–K^+^ electrostatic force keeps the potassium ions far away from the reference K^+^ so that the short-range van der Waals force is vanishing. Other trends can easily be identified, but they are more difficult to interpret. For instance, the van der Waals force exerted by Cl^−^ ions always pushes the reference K^+^ ion towards the graphene pore. Moreover, the van der Waals force exerted by water molecules, at intermediate distance from graphene pulls the reference K^+^ ion into the bulk, but it pushes it towards the pore at closer distances from the graphene layer. The pattern exhibited by the chloride and water van der Waals force is discussed in more detail in the next section. As a final note, we can now explain the behaviour of the total van der Waals force. In particular, the reversal in the direction of this force occurs when the repulsive force exerted by graphene becomes dominant with respect to the force exerted by water that tends to push the reference K^+^ ion towards the pore.

### Water and chloride van der Waals forces

The pattern exhibited by the van der Waals force of water can be explained in terms of the several factors. First of all, the analysis of the Lennard-Jones (LJ) potential of the potassium/water-oxygen and potassium/water-hydrogen interactions (ESI Fig. SF1 and SF2†) reveals that K^+^ has negligible Lennard-Jones interactions with the hydrogen atoms belonging to water molecules of both the first and second shell. This is because the peak of the first two shells (determined from the RDF profiles) corresponds to the flat tail of the Lennard-Jones curve. For the same reason K^+^ experiences vanishing Lennard-Jones forces from the oxygen atoms belonging to the water molecules of the second shell. Conversely, potassium is subject to repulsive forces from the oxygen atoms of the first water shell because the peak of this shell is located in the repulsive branch of the LJ potential.

A second ingredient potentially affecting van der Waals forces is the peak in water density that may be expected near the graphene since there will be adsorption of a water surface layer and a depletion of water in the pore. Indeed, the water number density profile as a function of *z*, shown in ESI Fig. SF3,† displays high peaks on either sides of the graphene sheet. The analysis of Lennard-Jones potential, however, suggests that these water density peaks can determine maxima of the LJ force only if they result in a significant increase in the number of water molecules of the first shell (the interaction with water molecules in higher order shells is negligible). Since the steric effect exerted by the graphene wall is expected to affect mainly the closest water molecules, it is convenient to divide each hydration shell in two semi-shells. The semi-shell that is on the same side as the graphene layer with respect to the K^+^ ion will be dubbed proximal while the other semi-shell will be called distal. In order to test whether the water density peaks affect the composition of the first shell, in ESI Fig. SF4† we show the profiles of the number of water molecules in the proximal and distal semi-shells of the 1-st shell. It can be noted that while the proximal semi-shell becomes water depleted (due to the steric hindrance of the graphene layer), the number of water molecules in the distal semi-shell remains constant or increases only slightly. The data therefore suggest that the composition of the first shell is basically unaffected by the water density peaks. It may be presumed that the water density peaks can result in an increase in the number of water molecules of the second and higher order shells, that however, are so far from the K^+^ ion to exert only negligible LJ forces. The origin of the peaks of the van der Waals forces is thus related to the selective loss of water in the proximal semi-shells. This leaves in the distal semi-shell an excess of water molecules that push the K^+^ ion towards the graphene sheet. Conversely, when the K^+^ ion is at intermediate distance from graphene, the electric field generated by the charged pore induces the reorientation of part of the water molecules of the proximal semi-shell of the first shell. As a result, the proximal semi-shell features an excess of water molecules with oxygen oriented towards K^+^. Since these oxygen atoms have been shown to exert a repulsive LJ force, they push the K^+^ ion away from graphene and into the bulk phase.

A similar approach can be used to explain the pattern of the van der Waals force exerted by chloride ions. Analysis of the Lennard-Jones potential (ESI Fig. SF5†) shows that K^+^ does not interact with the chloride ions belonging to the second coordination shell. Conversely, K^+^ has repulsive interactions with the Cl^−^ ions of the first shell. When the K^+^ ion is in the middle of the bulk, far from graphene, the Cl^−^ ions are symmetrically distributed in the 1-st shell so that the repulsive forces tend to balance each other. When the K^+^ ion comes close to graphene there will be a preferential loss of Cl^−^ ions from the proximal side of the first shell (ESI Fig. SF5†). As a result, the first shell becomes asymmetrical, with an excess of chloride ions on the side distal to graphene. Since these Cl^−^ ions exert a repulsive LJ force, they push the K^+^ ion towards graphene in agreement with the plots in Fig. 9 and 10.

### Analysis of chloride permeation

**Fig. 11:**
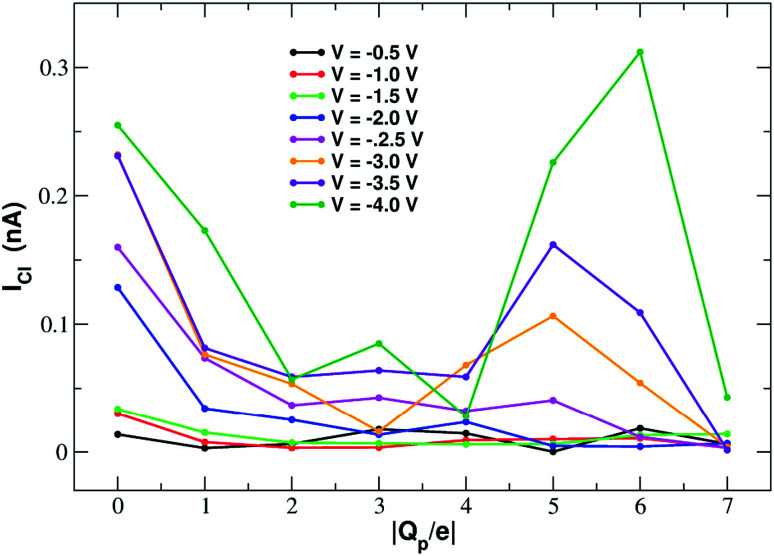
Chloride current as a function of the pore charge. The calculation has been repeated at voltages from −0.5 V to −4.0 V at 0.5 V intervals.

Fig. 11 shows that the variation of chloride current with pore charge presents an irregular behaviour where an initial decrease up to *Q*_p_ = −2*e* is followed by a plateau stage up to *Q*_p_ = −4*e* and then a new increase at *Q*_p_ = −5*e* before falling again at *Q*_p_ = −6*e* and −7*e*. The initial decrease of the current is due to a polarization effect in the ion concentration that will be discussed in more detail when analyzing the PMFs profiles. The peculiar behaviour in the other regions of the curve is due to the fact that the small pore size results in a high density of negative charge. This in turn, at rim charge higher than 4*e*, attracts a cluster of K^+^ ions immediately below the graphene layer. These potassium ions screen the negative charge of the pore and allow larger chloride currents. When the pore charge equals −7*e*, however, there are so many potassium ions in the cluster below the graphene layer that they strongly attract the chloride ions preventing them from crossing the pore.

In order to explain this behaviour we plot the number density of potassium and chloride ions in axial bins with height 0.5 Å and radius 4.5 Å (equal to the pore radius) choosing as an example the simulation at −4.0 V. The potassium profile in ESI Fig. SF6(a)† shows a main peak at *z* = −5.0 Å that slowly increases and shifts closer to the graphene pore as the rim charge increases from *Q*_p_ = 0 to *Q*_p_ = −4. Further increase of the charge, however, causes the appearence of a second extremely high density peak at approximately *z* = −2.0 Å. This pattern can easily be explained. As long as the charge is not too high, potassium ions are localized below the graphene sheet (because the electric field pushes them downwards) and close to the pore but not so close as to exit from the bulk phase and lose their hydration shells. On the other hand, when the pore charge becomes very high, the coulombic attraction is so strong that a close contact with the charged pore becomes more energetically convenient than remaining in the bulk solution to maintain the hydration energy.

The behaviour of the chloride density profile (ESI Fig. SF6(b)†) is strongly affected by the distribution of K^+^ ions. When *Q*_p_ = 0 there is a single chloride density peak at *z* = −4.0 Å. This is due to the fact that the electric field is oriented towards the negative *z*-axis. This means that the negatively charged chlorides are pushed towards the positive *z*-axis. However, since graphene is not very permeable to chloride, they tend to accumulate just below the pore. As the rim charge is increased up to *Q*_p_ = −4, the chloride peak decreases and shifts further away from the pore at *z* = −5.0 Å due to the increasing electrostatic repulsion exerted by the pore. However, when the charge is increased to *Q*_p_ = −6 and −7 there will be the appearance of three high density peaks at *z* = −7.0 Å, *z* = −5.0 Å and *z* = −2.0 Å. This behaviour is clearly a consequence of the build-up of two high potassium density peaks close to the pore. The high local potassium concentration screens the negative charge of the pore and the K^+^/Cl^−^ attraction allows the appearance of a high Cl^−^ concentration. Moreover, the shielding of the pore charge allows an increase of the Cl^−^ current as shown in the current plot (Fig. 11) at *Q*_p_ = −5 and *Q*_p_ = −6. The drop of the Cl^−^ current observed at *Q*_p_ = −7 is due to the fact that the K^+^/Cl^−^ attraction has become so strong that the chloride ions are trapped in electrostatic cages of potassium ions.

ESI Fig. SF7† shows the current–voltage relation of chloride currents at different values of the pore charge. Even if the curves are irregular, it can be noted that for small voltages they remain constant or grow very slowly but, after a threshold voltage is passed, the slope of the curve increases abruptly. The threshold voltage becomes larger and larger as the pore charge is increased. This phenomenology suggests a scenario whereby the chloride ion needs to overcome a very high permeation barrier and only when the applied voltage allows the jump over the barrier a significant current can ensue.

**Fig. 12:**
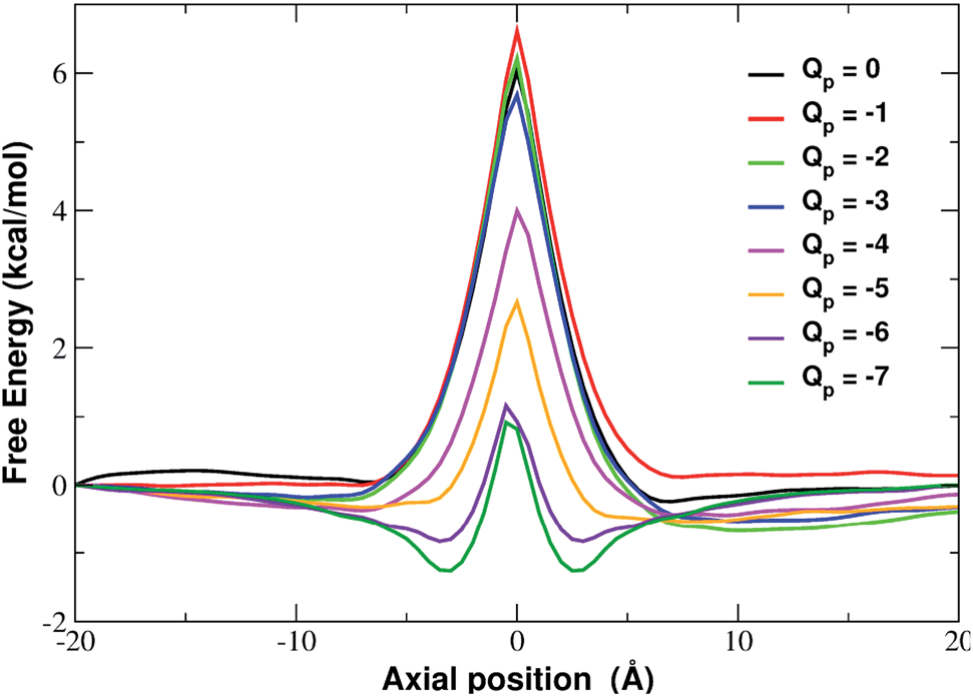
Potential of mean force of chloride as a function of the axial position. The calculation has been repeated for different charged systems with pore charge ranging from *Q*_p_ = 0 to *Q*_p_ = −7. Charges are expressed in elementary charge units.

More insight into the permeation mechanism of chloride can be attained from the analysis of the PMF profiles. The PMF was again computed through umbrella sampling simulations using a protocol identical to the one employed for the calculation of the K^+^ PMF except that each window was run for 16 ns instead of 6 ns to reach convergence. The first nanosecond of the simulations was discarded as equilibration and the analysis was performed on the remaining 15 ns. The PMF profiles appearing in Fig. 12 show a high free energy barrier due partly to desolvation and partly to electrostatic repulsion from the charged rim. The barrier height remains approximately constant as the charge is increased from *Q*_p_ = 0 to *Q*_p_ = −3 and then drops from *Q*_p_ = −4 to *Q*_p_ = −7. This behaviour is seemingly at odds with the current/charge plots shown in Fig. 11. In fact, the current plots show a significant drop of the current when the charge is increased from *Q*_p_ = 0 to *Q*_p_ = −1 and *Q*_p_ = −2 that does not seem to be justified by the very similar peaks of the PMF profiles. A clue to help in disentangling this contradiction is provided by the current plot in Fig. 11. Note that the current drop when passing from *Q*_p_ = 0 to *Q*_p_ > 0 becomes more and more pronounced as the applied voltage increases. This suggests that the high current observed at *Q*_p_ = 0 could be due to some peculiar ion distribution induced by the external electric field. To test this hypothesis in ESI Fig. SF8† we computed the number density profiles of K^+^ and Cl^−^ ions in the simulation with an applied potential of −4.0 V. It can be noticed that at *Q*_p_ = 0 the ion distribution around the pore is highly asymmetrical. Specifically, the Cl^−^ concentration below the pore is much higher than the concentration of the same ion above the graphene layer, while the potassium concentration above the pore is only slightly higher than the K^+^ concentration below the pore. This distribution determines that, in the neighborhood of the pore, there is a large availability of chloride ions that could be involved in permeation. This results in a high chloride current even if the free energy barrier of the PMF is high. It must be stressed explicitly that, since the PMF was computed in absence of external field, this concentration polarization effect could not be accounted for by the PMF profiles. As a comparison, we also computed the ion density profiles for the system with pore charge *Q*_p_ = −2 in the presence of an external potential of −4.0 V. In this case it can be observed that the chloride density below the pore is only moderately higher than that above the pore. Thus, the gradient of concentration of Cl^−^ is smaller than in the *Q*_p_ = 0 case explaining why the current at *Q*_p_ = −2 is much smaller than that at *Q*_p_ = 0 even though the peaks of the PMFs are almost identical. As a final note, it can be observed that the shallow free energy wells appearing on either side of the barrier at *Q*_p_ = −6 and *Q*_p_ = −7 are also due to the high local density of K^+^ ions that attract the incoming chloride.

## Conclusions

One of the many potential applications of graphene concerns water desalination and purification. While the sieving capability of small graphene pores depends on steric effects, the selectivity of larger pores critically depends on the charge. Yet, only a couple of studies systematically addressed the issue of selectivity/charge relationship. Zhao *et al.*^16^ showed that even large graphene nanopores, if negatively charged, can enhance K^+^ transport while completely rejecting Cl^−^. Even if this work explores a wide range of values of pore charge and radius, no attempt was made to study the influence of the charge on potassium and chloride PMFs. Similarly, Li *et al.*^37^ recently showed that a charged graphene surface can induce selectivity of Li^+^ and Na^+^ over K^+^. However, these authors, instead of analyzing classical pores generated by removal of carbon atoms, considered ion flow through the stretched aromatic rings of a graphene membrane under mechanical strain.

Our systematic exploration of the influence of the pore charge on both the *I*/*V* curves and the PMF profiles fills a gap in the scientific literature. As a cautionary note, it must be observed that we chose to use a simplified pore charge distribution (negative charge evenly spread on rim atoms and positive neutralizing charge evenly spread on the other carbon atoms). In realistic functionalized graphene nanopores the charge distribution can be different. Remarkable examples include the bio-inspired nanopores studied by Kang *et al.*^21^ and the graphene-based crown ethers analysed by Guo *et al.*^38^ and by Smolyanitsky *et al.*^39^ where the electrostatic potential maps critically depend on the placement and orientation of the ether dipoles. Despite its simplicity however, our approach can qualitatively reproduce the properties of realistic systems. This is due to the fact that many patterns are significantly robust with respect to the fine details of the charge distribution. As an example, Zhao *et al.*^16^ who used a charge model similar to ours, showed that, as long as the total charge is kept constant, charging all pore atoms or only alternating ones led to comparable ion fluxes. Similarly to what is reported in ref. 37 about Li^+^, we found that, as the pore charge is increased, the barrier of K^+^ PMF decreases until it turns into a well that partially traps potassium, reducing the current. This trend can be explained in terms of an increasing electrostatic compensation of a roughly constant desolvation cost. This is in agreement with the seminal theory developed by Eisenman^26,27^ that identifies two driving forces for ion permeation: (i) the ion–pore electrostatic interactions and (ii) the desolvation cost the ion incurs when crossing the pore. More formally, an ion with charge *q* and radius *r* has a hydration free energy represented by the Born approximation: *G*_hydr_ = *q*^2^/8π*ε*_w_*r* where *ε*_w_ is the dielectric constant of water and its electrostatic interaction with a site within the channel of charge *q*_s_ and radius *r*_s_ is *G*_int_ = *qq*_s_/4π*ε*_w_(*r* + *r*_s_). Ion permeation can only occur if *G*_int_ ≥ *G*_hydr_. It is noteworthy that use of the Born formula to express the dehydration cost, amounts to assuming that the ion is completely desolvated upon entering the pore. Eisenman's ideas were further developed by Zwolak and coworkers^28,29^ who expressed the desolvation cost as a function of the pore radius. This approach accounts for partially desolvated states since dehydration occurs in a quantized way, a shell at a time. According to this model desolvation only depends on the geometric properties of the pore, namely its radius. Our results however, show that this picture is oversimplified. Even if the large number of water molecules lost by the ion upon entering the neutral pore confirms the importance of geometry, Fig. 6 shows that pore charge enhances the loss of water in the first shell. Ref. 37 shows that this trend is true not only for K^+^ but also for Na^+^ and Li^+^.

The decrease of the free energy barrier as a function of increasing pore charge leads to the issue of near-barrierless permeation. In the literature it is widely debated whether barrierless conduction must be considered a universal mechanism of ion channels or a peculiar property of KcsA-like channels where two- and three-ion occupation states are isoenergetic. Yesylevskyy and Kharkyanen^40^ on the grounds of theoretical modelling and Brownian dynamics simulations suggested that knock-on barrierless conduction can be considered as a general mechanism of transport in ion channels with multiple occupancy. Similar conclusions have been reached by the Ionic Coulomb Blockade model^41^ predicting that conduction bands occur when the charge of the selectivity filter balances the charge of the ions already inside the channel plus the image charge of a further potentially incoming ion. Our simulations show that barrierless conduction can be tuned using pore charge as an adjustable parameter. In fact, when the rim charge is high enough, the ion/pore interaction almost perfectly balances the desolvation cost flattening the permeation barrier. Recent computational studies have shown that barrierless conduction can be induced using other control parameters. For instance Fang *et al.*^42^ showed that a modest mechanical strain on graphene-embedded crown ether pores reduces the K^+^ release barrier, significantly increasing the current. Interestingly, in this case also, the barrier arises from both the desolvation cost and ion–pore electrostatics. The mechanical pore expansion flattens the barrier simultaneously weakening the ion/pore interactions and strengthening the ion/water ones.

The atomistic level insight provided by molecular dynamics not only sheds light on the details of the desolvation process, but it also reveals a rich picture not foreseen by simplified physical models. Our simulations show that van der Waals and electrostatic forces exert opposite effects on the permeation process (Fig. 8). For small values of the pore charge van der Waals forces tend to push the K^+^ ion towards the pore while electrostatic forces tend to keep it in the bulk. However, as the pore charge is increased, the directions of both forces are reversed. The reversal of the van der Waals force is due to the build-up of an increasing repulsive force exerted by graphene that becomes dominant over the LJ forces exerted by chloride and water. The reversal of the electrostatic force depends on two main factors. First of all, as pore charge increases, the attractive electrostatic force exerted by graphene overcomes the repulsive force exerted by water. The interplay between these two forces is the one expected based on simplified models like Eisenman's and Zwolak's. The simulations however, also reveal an important but unexpected role of potassium and chloride ions. For small values of the pore charge these ions are evenly spread in the bulk phase so that their forces (the reference K^+^ ion is attracted to the bulk by chloride and pushed to graphene by the other potassium ions) tend to balance each other. At high pore charges however, the formation of a concentration polarization layer of K^+^ ions switches the sign of the electrostatic force that potassium ions exert on the reference K^+^ ion. The formation of concentration polarization layers is even more significant in the case of Cl^−^ permeation reconciling the seeming mismatch between the modest decrease of the barrier of the chloride PMF (when *Q*_p_ varies from 0 to −3) and the significant drop of the current.

Concentration polarization, the non-uniform ion distribution close to a sufficiently small pore is a widespread phenomenon also highlighted in other studies. For instance Rollings *et al.*^43^ observed a surprisingly large K^+^/Cl^−^ selectivity in pores even as large as 20 nm in diameter. A careful examination of the results attributed this counter-intuitive result to the elevated concentration of mobile cations near the electronegative graphene surface. More recently, Hu *et al.*^24^ showed through MD simulations that sodium and potassium form concentration polarization layers on either side of a graphene sheet. In their work with a neutral pore, Na^+^ forms symmetrical density peaks on either side of the graphene pore similarly to what happens with K^+^ in our simulation with *Q*_p_ = 0. Chloride, on the other hand, forms a single very high density peak just above the membrane. This mismatch with our simulations where the Cl^−^ density peak is below the pore, is simply due to the different orientation of the electric field in their work.

In summary, we have characterized the influence of pore charge on the permeation of potassium and chloride across a graphene nanopore. While the simulations basically confirm the validity of simplified physical models, they also reveal a much richer phenomenology where the asymmetry of ion distributions and the orientational effects induced by the applied potential and pore charge significantly affect conduction, thus calling for more detailed and comprehensive modelling.

## Conflicts of interest

There are no conflicts to declare.

## Supplementary Material

RA-009-C9RA03025E-s001
