# Supplementary material for: Exploring the pore charge dependence of K+ and Cl− permeation across a graphene monolayer: a molecular dynamics study
Source: RSC Adv. 2019 Jul 1;9(35):20402–14. doi: 10.1039/c9ra03025e (PMC9065459; doi:10.1039/c9ra03025e)
Supplement: RA-009-C9RA03025E-s001 [file RA-009-C9RA03025E-s001.pdf]

Electronic Supplementary Material (ESI) for RSC Advances.
This journal is © The Royal Society of Chemistry 2019

ARTICLE TYPE

Exploring the pore charge dependence of K^+ and Cl^- permeation across a graphene monolayer: a Molecular Dynamics study †

Carlo Guardiani,[a*] William A.T. Gibby,[a] Miraslau L. Barabash[a], Dmitri G. Luchinsky[a,b] and Peter V.E. McClintock[a]

SUPPORTING INFORMATION

[a] *Department of Physics, Lancaster University, Lancaster LA1 4YB, UK*
[b] *SGT Inc., Greenbelt, MD, 20770, USA*
[*] Corresponding author. Fax: +44 (0) 1524 844037; Tel: +44 (0)1524 593073;
E-mail: c.guardiani@lancaster.ac.uk
† Electronic Supplementary Information (ESI) available: 8 Supporting Figures.

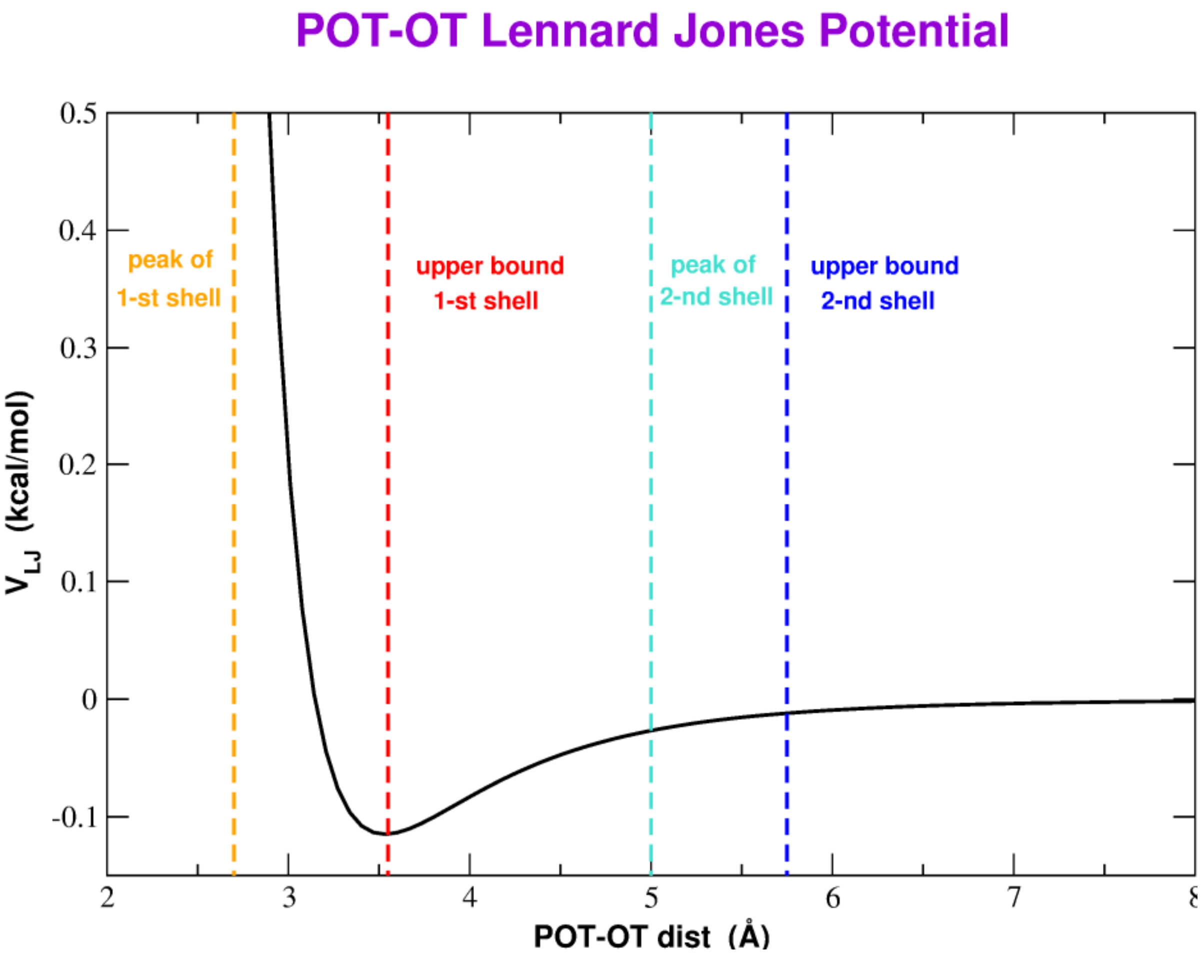

Fig. 1 Lennard Jones potential for the interaction between a potassium ion and a water oxygens. Parameters have been taken from the CHARMM27 force field. The orange and cyan dashed lines mark the position of the first two peaks of the K^+/water-oxygen Radial Density Function (RDF). The red and blue dashed lines indicate the position of the first two minima of the RDF that we use as delimiters of the first two hydration shells of K^+.

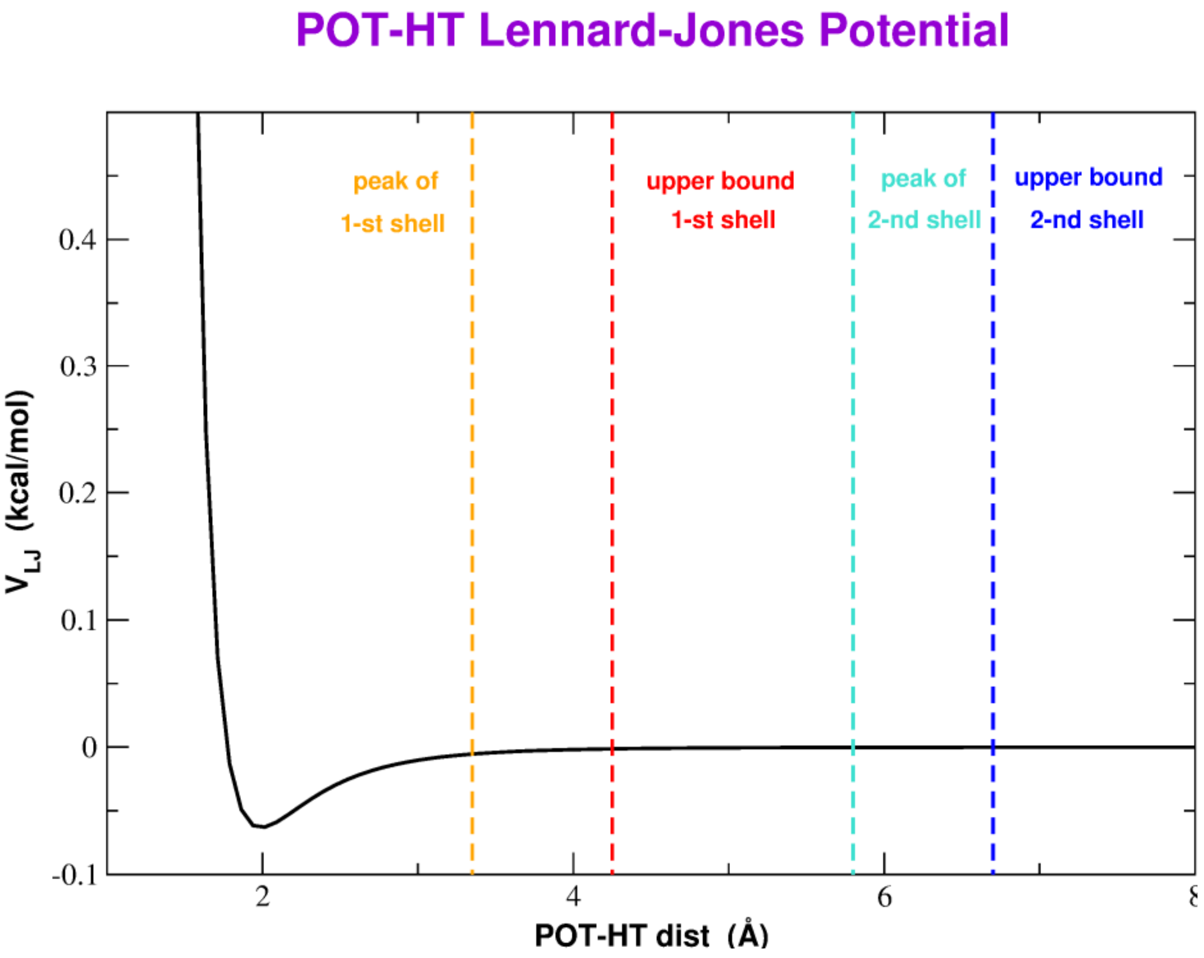

Fig. 2 Lennard Jones potential for the interaction between a potassium ion and a water hydrogens. Parameters have been taken from the CHARMM27 force field. The orange and cyan dashed lines mark the position of the first two peaks of the K^+/water-hydrogen Radial Density Function (RDF). The red and blue dashed lines indicate the position of the first two minima of the RDF that we use as delimiters of the first two hydration shells of K^+.

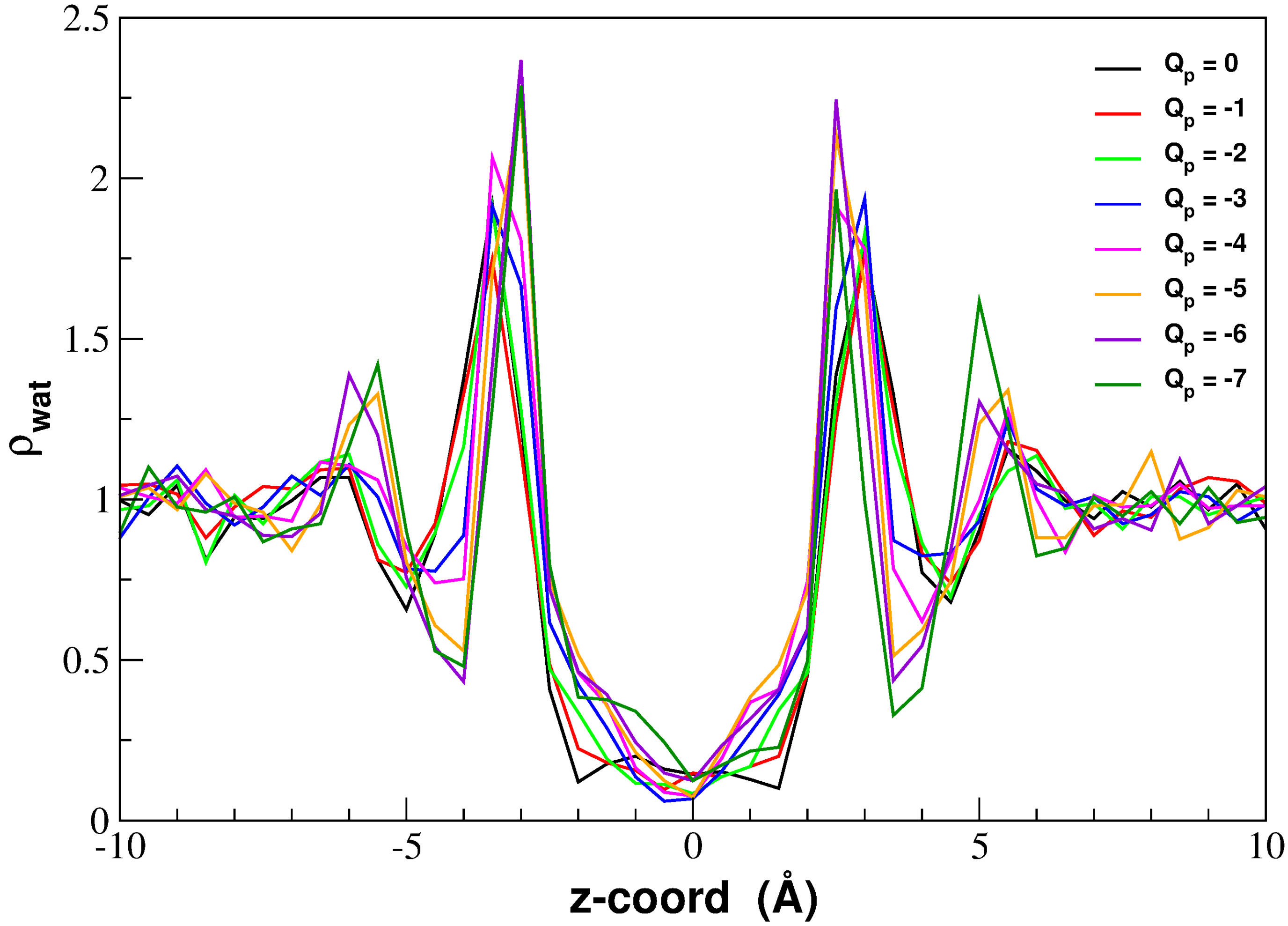

Fig. 3 Water number density profiles as a function of the axial position z in the Umbrella Sampling simulations with bias on a K^+ ion. Charges are expressed in elementary charge units.

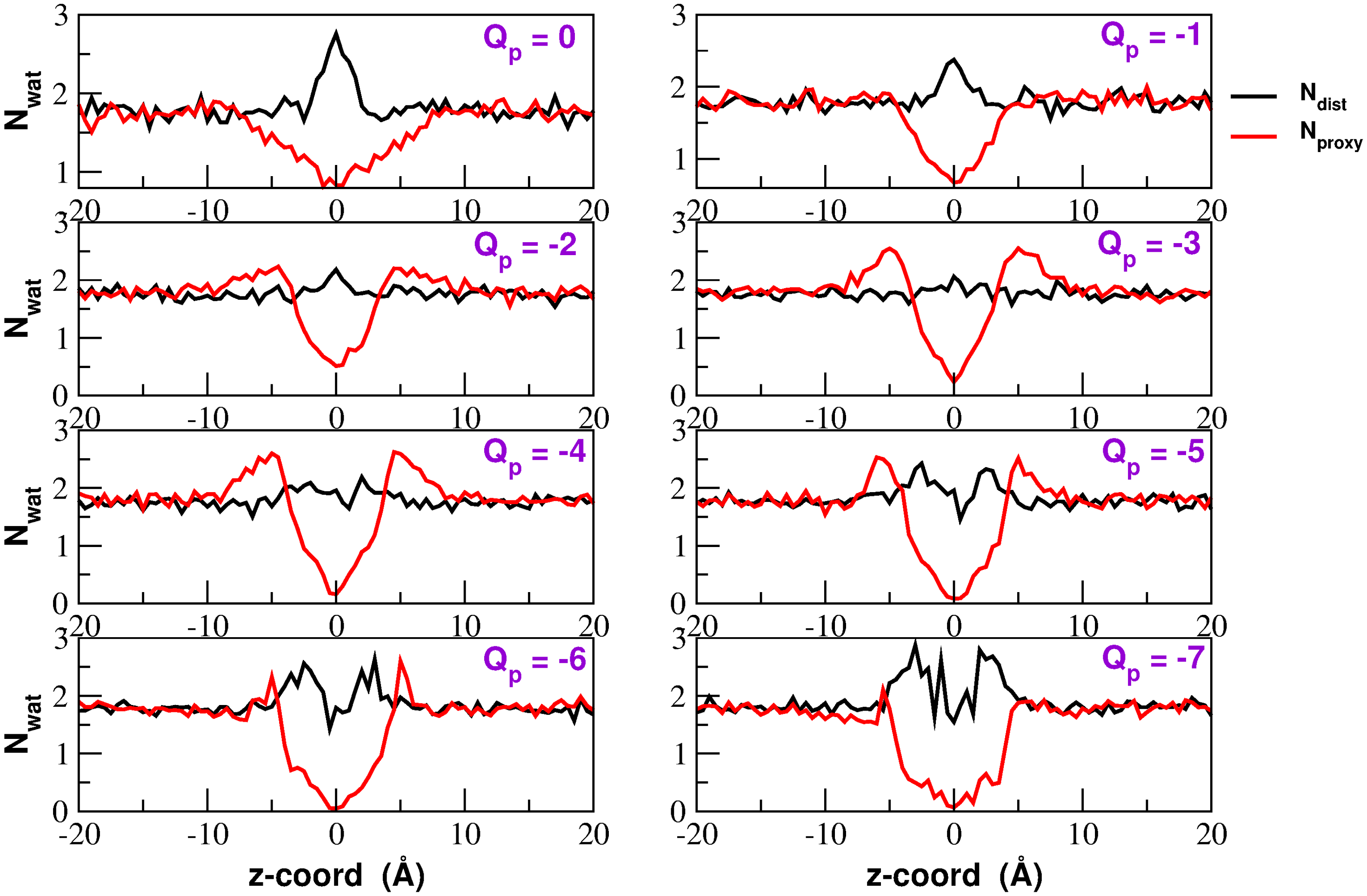

Fig. 4 Number of water molecules in the distal (black line) and proximal (red line) semi-shell of the first hydration shell of the K^+ ion biased in the Umbrella Sampling simulations, as a function of the axial position z. Charges are expressed in elementary charge units.

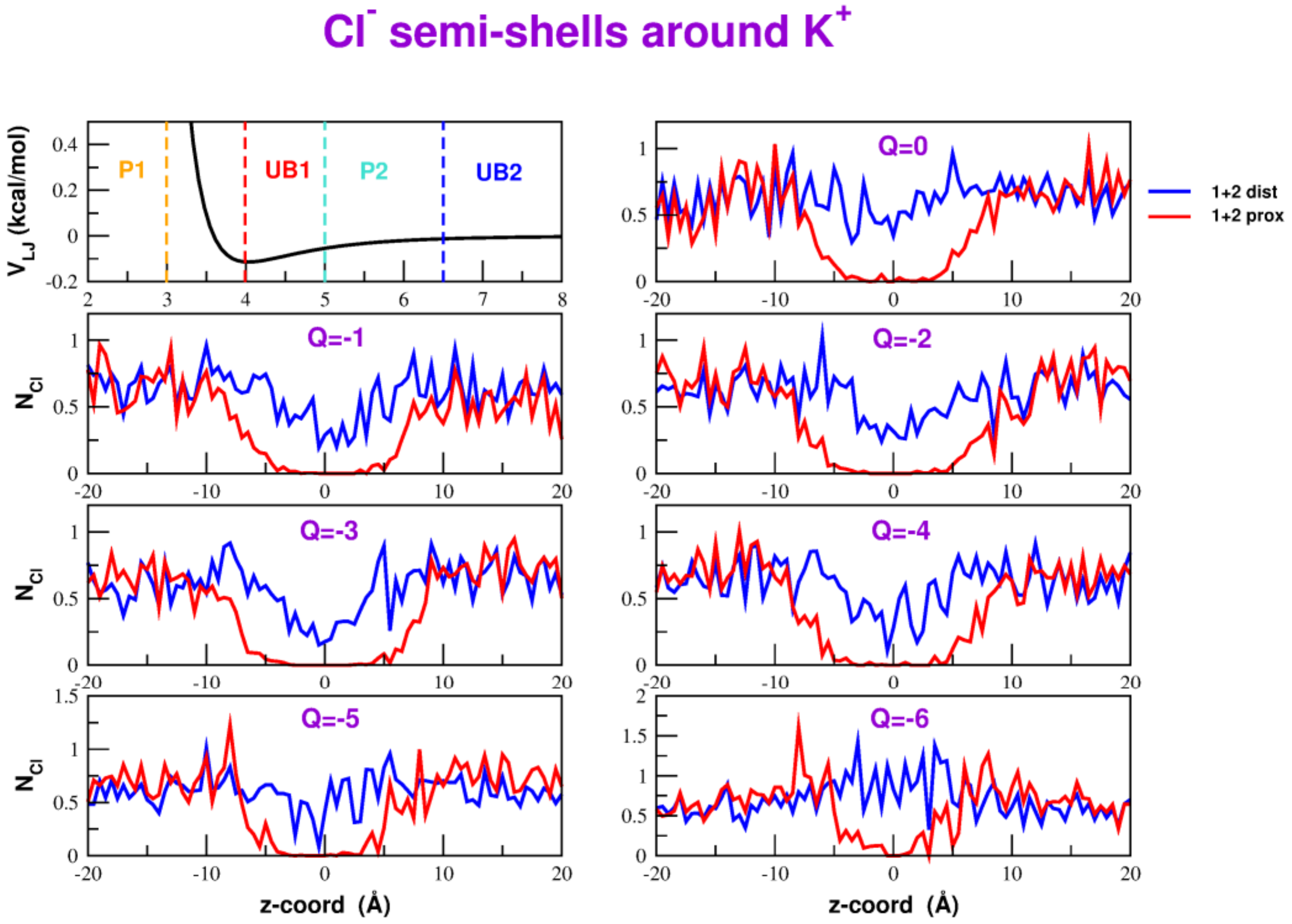

Fig. 5 Analysis of K^+-Cl^- van der Waals interactions. The top left panel shows the Lennard Jones potential for the interaction between potassium and chloride ions. Parameters have been taken from the CHARMM27 force field. The orange and cyan dashed lines mark the position of the first two peaks of the K^+/Cl^- Radial Density Function (RDF). The red and blue dashed lines indicate the position of the first two minima of the RDF that we use as delimiters of the first two coordination shells of K^+. The remaining panels show the number of water molecules in the distal (blue line) and proximal (red line) semi-shells of the first two Cl^- coordination shells of the reference K^+ ion. Charges are expressed in elementary charge units.

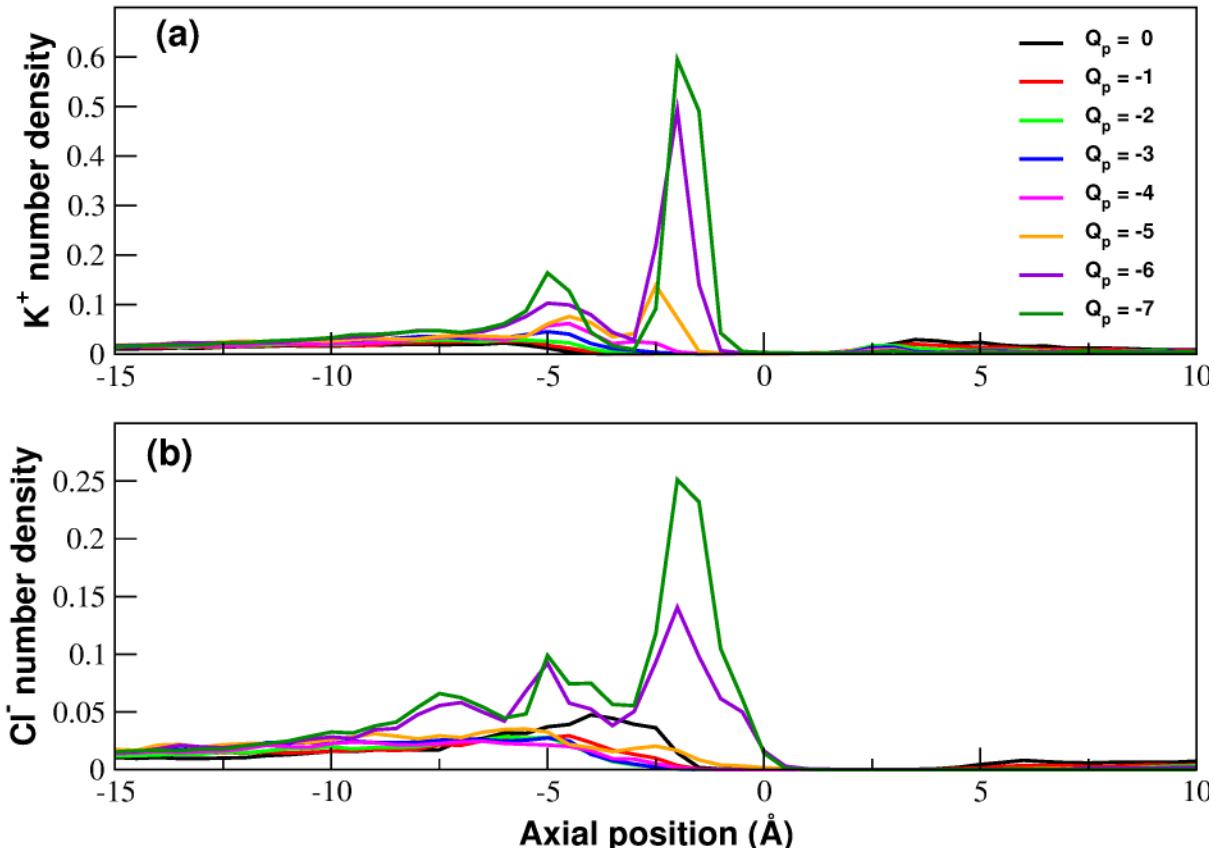

Fig. 6 Number density of potassium (a) and chloride ions (b) in the simulation with applied voltage of -4.0 V. Charges are expressed in elementary charge units. Number densities are computed as the number of potassium and chloride ions in axial bins with height 0.5 Å and radius 4.5 Å (equal to the pore radius).

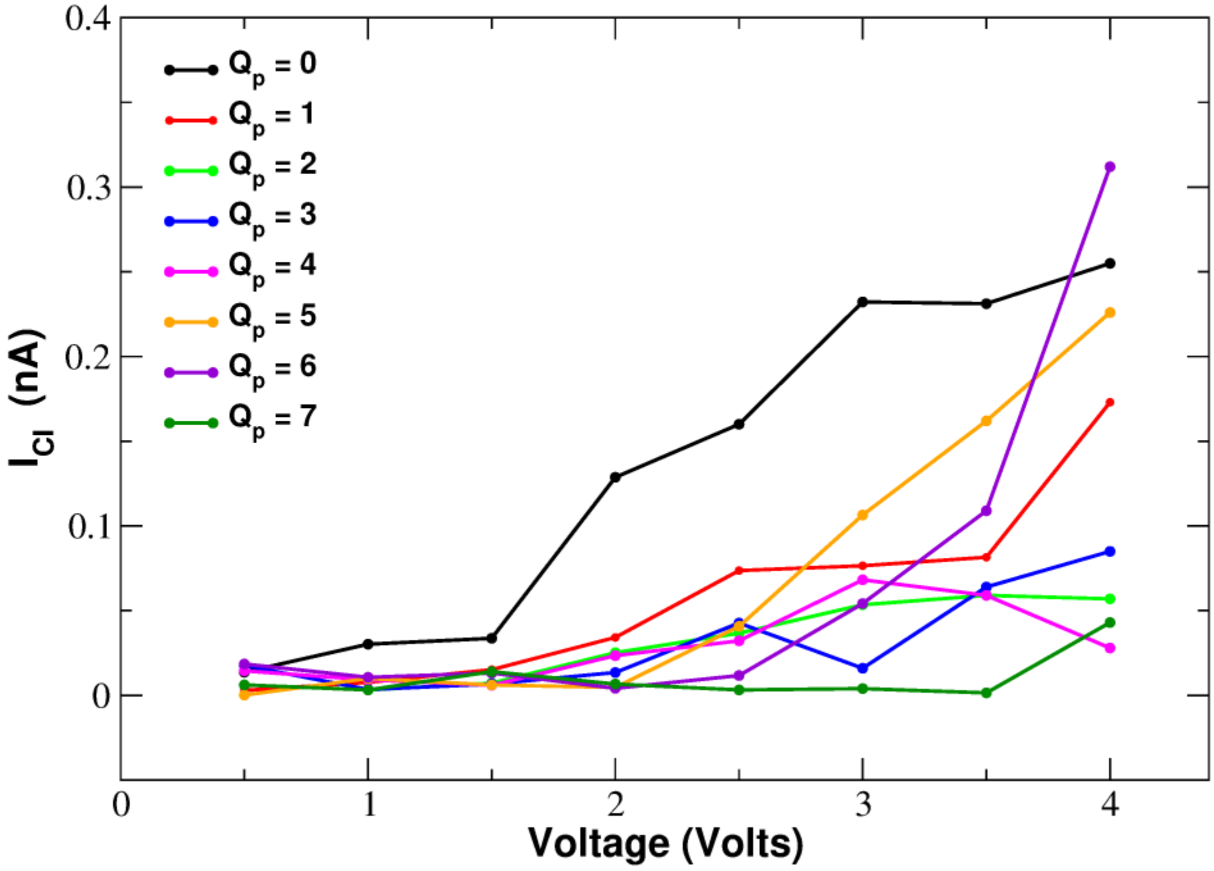

Fig. 7 Current-voltage relationships of chloride at different charges of the graphene pore. For graphical clarity the x-axis shows the absolute value of the negative voltages used in the simulations. Charges are expressed in elementary charge units.

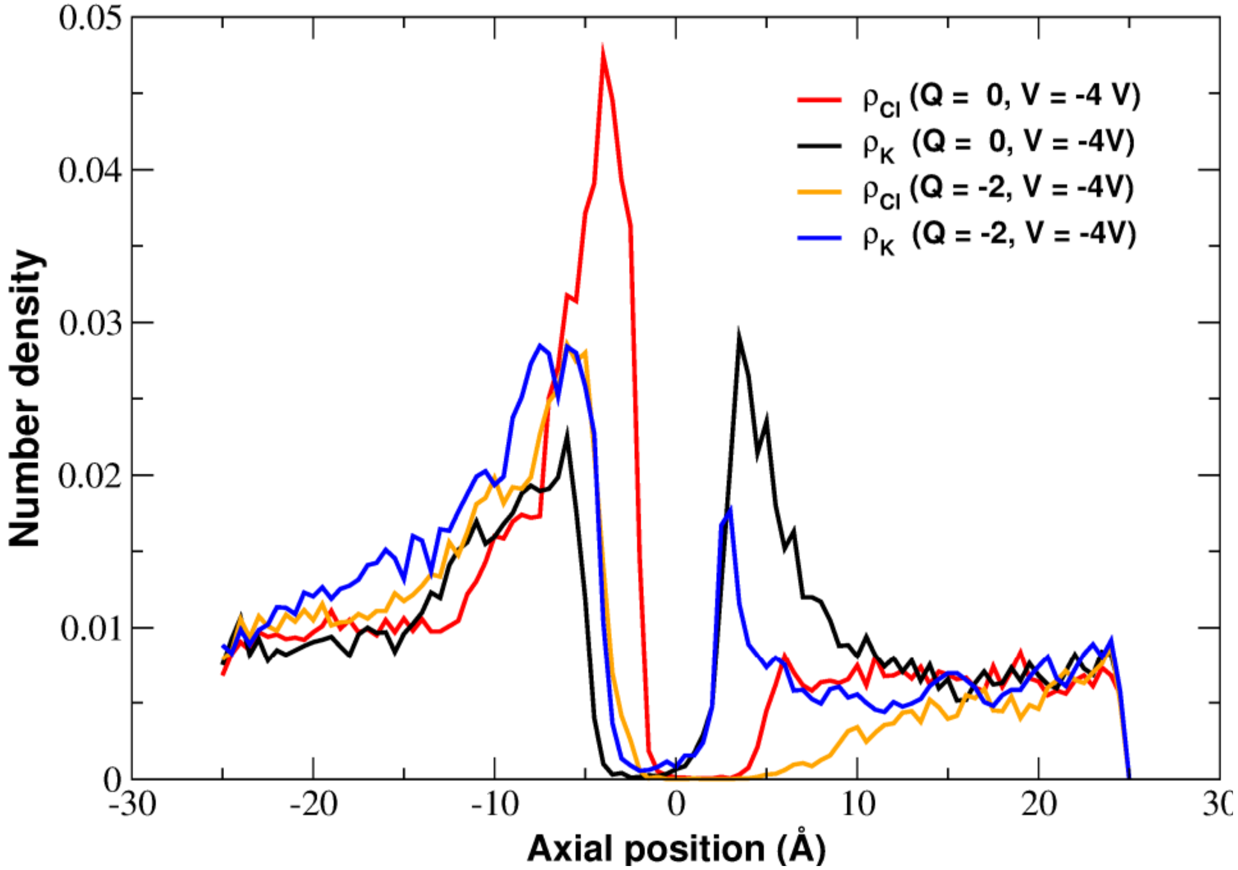

Fig. 8 Number density profiles of K^+ and Cl^- ions computed for the systems with pore charge Q_p=0 and Q_p=-2 in the presence of and applied potential of -4.0 V. Charges are expressed in elementary charge units. Number densities are computed as the number of potassium and chloride ions in axial bins with height 0.5 Å and radius 4.5 Å (equal to the pore radius).

Conflicts of interest

There are no conflicts to declare.

Acknowledgements

The authors gratefully acknowledge financial support from the Engineering and Physical Sciences Research Council (grants EP/M016889/1 and EP/M015831/1), and by a Leverhulme Trust Research Project Grant RPG-2017-134.
